# Supplementary material for: Identification of preclinical dementia according to ATN classification for stratified trial recruitment: A machine learning approach
Source: PLoS One. 2023 Oct 19;18(10):e0288039. doi: 10.1371/journal.pone.0288039 (PMC10586674; doi:10.1371/journal.pone.0288039)
Supplement: S1 Appendix — (DOCX) [file pone.0288039.s001.docx]

**S1 Appendix**

## **Study assessments tables**

Table A: Construction of the different levels (0-4) of the Smoking score (ever smoked) Framingham I variable.
Legend: Ever smoked: 1 – yes, 0 – no; Female: 1 – female, 0 – male.

| Ever smoked | Age bands | Female | Smoking score (ever smoked) Framingham I |
| --- | --- | --- | --- |
| 1 | 50/54 | 1 | 4 |
| 1 | 55/59 | 1 | 4 |
| 1 | 60/64 | 1 | 2 |
| 1 | 65/69 | 1 | 2 |
| 1 | 70/74 | 1 | 1 |
| 1 | 75+ | 1 | 1 |
| 0 | any | 1 | 0 |
| 1 | 50/54 | 0 | 3 |
| 1 | 55/59 | 0 | 3 |
| 1 | 60/64 | 0 | 1 |
| 1 | 65/69 | 0 | 1 |
| 1 | 70/74 | 0 | 1 |
| 1 | 75+ | 0 | 1 |
| 0 | any | 0 | 0 |

Table B: Description of the different levels (0-5) of the categorical variable Physical activity.

| Physical activity (ordinal) | 0 | 1 | 2 | 3 | 4 | 5 |
| --- | --- | --- | --- | --- | --- | --- |
| Meaning of the level | Not at all | A few times a year | 2-3 times a month | Once a week | 2-3 times a week | Daily |

**Features Inclusion in Machine Learning Models**

Table C shows the inclusion of the different features in the optimal models with 7, 10 and 13 features for EPAD and ADNI data. Each (double) row represents a feature, each column represents a comparison. Non-shaded cells indicate the presence of the particular feature in the optimal models for the EPAD dataset, shaded ones – for the ADNI dataset. Numbers (7, 10 or 13) represent number of features, abbreviations (rf, sv) represent random forest and linear-kernel SVM respectively. Each cell is either blank or consists of one or more combinations of number of features and type of ML model. If such combination is present in a particular cell, this indicates that the feature, corresponding to the row is part of the optimal model with the particular number of features of the particular type of ML method for the comparison that correspond to the column.

For example, the value of 10rf, 13rf in the non-shaded cell corresponding to row BMI and column Alzheimer’s and concomitant non-Alzheimer’s pathologic change vs Normal AD biomarkers indicate that the feature BMI is part of the optimal random forest models with 10 and 13 features for the Alzheimer’s and concomitant non-Alzheimer’s pathologic change vs Normal AD biomarkers comparison using EPAD data, but is not part of the optimal random forest model for that comparison with 7 features, nor of any of the linear-kernel SVM models for that dataset. Empty cell, e.g. the shaded one corresponding to row BMI and column Alzheimer’s and concomitant non-Alzheimer’s pathologic change vs Normal AD biomarkers indicate that the feature BMI is not part of any optimal model for that comparison for the ADNI dataset.

Table C: Features participation in the optimal models. Description of the abbreviations is given in the text above the table.

|  | Alzheimer’s pathologic change vs Normal AD biomarkers | Alzheimer’s disease vs Normal AD biomarkers | Alzheimer’s and concomitant non-Alzheimer’s pathologic change vs Normal AD biomarkers | Non-AD pathologic change  vs Normal AD biomarkers |
| --- | --- | --- | --- | --- |
| Age (years) | 7sv, 10rf | 7rf, 10rf, 13rf | 7rf | 7rf, 10rf, 13rf |
|  | 10sv | 10rf |  | 10sv |
| Female | 7sv, 10rf | 7rf, 10rf | 10rf, 13rf | 7rf, 10rf, 13rf |
|  |  | 10rf |  | 10sv |
| APOE4 (presence) | 7sv, 10rf, 13rf | 7rf, 10rf, 13rf | 10rf, 13rf | 13rf |
|  | 10sv | 10rf |  | 10sv |
| Dementia family history (yes) | 7sv, 10rf, 13rf | 7rf, 10rf, 13rf | 10rf, 13rf | 13rf |
|  |  | 10rf |  | 10sv |
| BMI |  | 7rf, 10rf, 13rf | 10rf, 13 rf | 10rf,13rf |
|  |  | 10rf |  | 10sv |
| White matter lesion volume | 7sv, 13rf | 7rf, 10rf, 13rf | 7 rf | 7rf, 10rf, 13rf |
|  | 10sv | 10rf |  | 10sv |
| MMSE total | 10rf, | 7rf, 10rf, 13rf | 10rf, 13 rf |  |
|  | 10sv | 10rf |  | 10sv |
| Education (years) | 10rf, 13rf | 10rf, 13 rf | 10rf, 13 rf |  |
|  |  | 10rf |  |  |
| RBANS semantic fluency |  |  | 10rf, 13 rf |  |
|  |  |  |  |  |
| Systolic blood pressure | 10rf |  | 10rf, 13 rf |  |
|  |  |  |  |  |
| RBANS delayed memory index |  |  |  | 13rf |
|  |  |  |  |  |
| Smoking score (ever smoked) Framingham I | 13rf | 13rf |  |  |
|  |  |  |  |  |
| Physical activity categorical |  |  |  | 13rf |
|  |  |  |  |  |

**Receiver Operating Characteristics (ROC)**

Figs A and B demonstrate the ROC curves for the EPAD dataset using 13 features and ADNI dataset using 10 features plus plasma tau respectively.


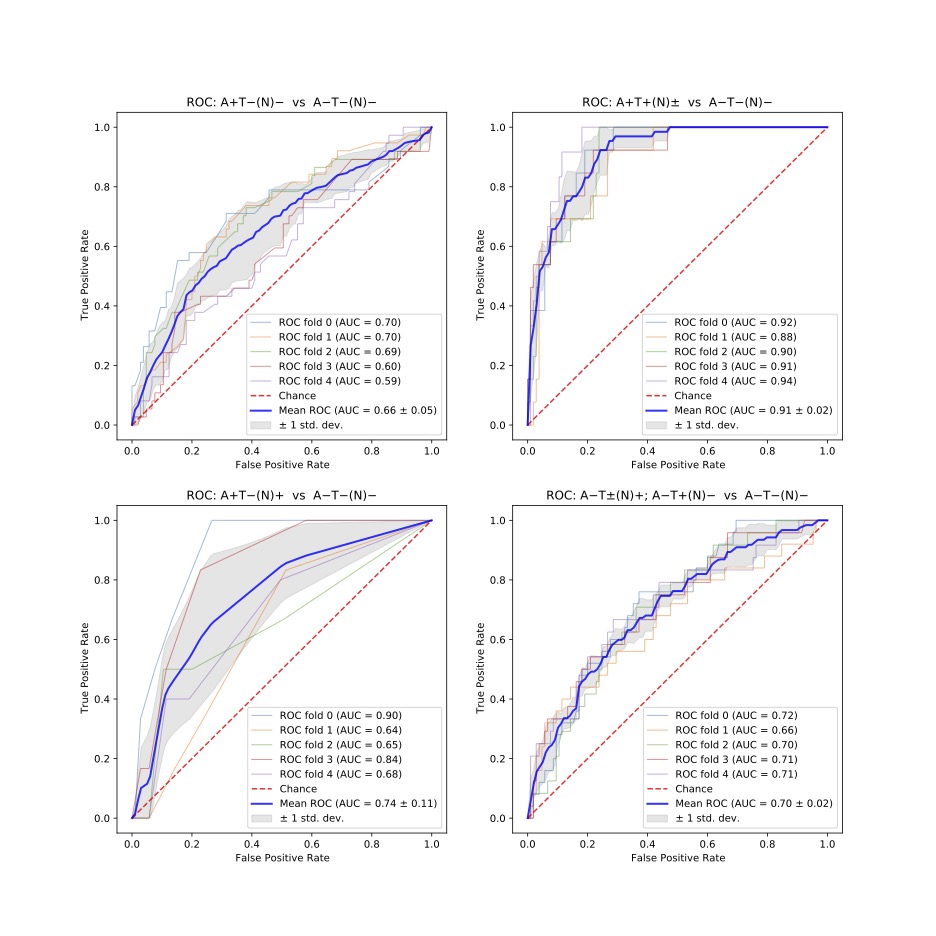


Figure A: ROC curves and AUC metrics for EPAD dataset using 13 features. Each pane shows an individual comparison as indicated on the top of the pane.


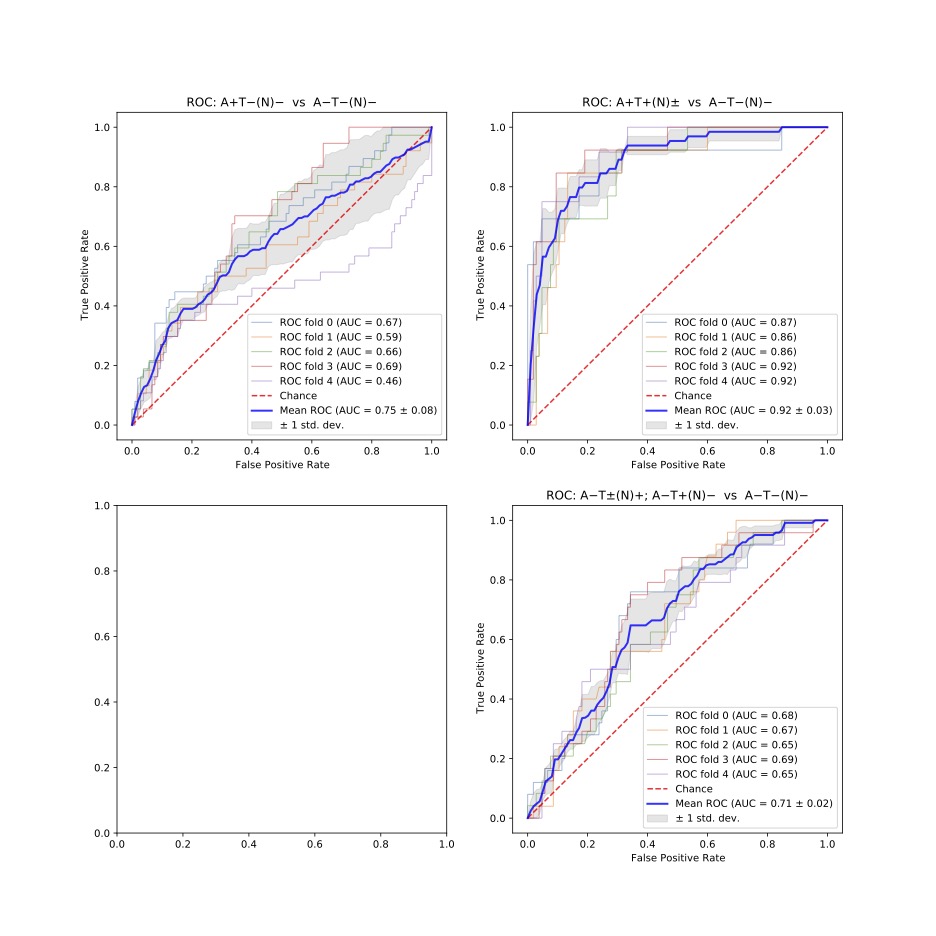


Figure B: ROC curves and AUC metrics for ADNI dataset using 10 features + plasma tau. Each pane shows an individual comparison as indicated on the top of the pane. Bottom-left pane is left blank due to insufficient sample size.

**Differences in Distribution between ADNI and EPAD**

The following graph shows quantile-quantile (Q-Q) plots for WHML of either the ADNI or the ADNI reduced dataset vs EPAD. Identical or close distributions would result in the Q-Q plot being on or close to the diagonal. The two panes show the Q-Q plots of the distributions of WHML of either ADNI or ADNI reduced dataset vs the one of EPAD. It is evident that the distributions are close for the left tails (smaller values of the distributions), and that they are very different for the right tails (larger values) of the distributions for which the plots diverge from the diagonal.


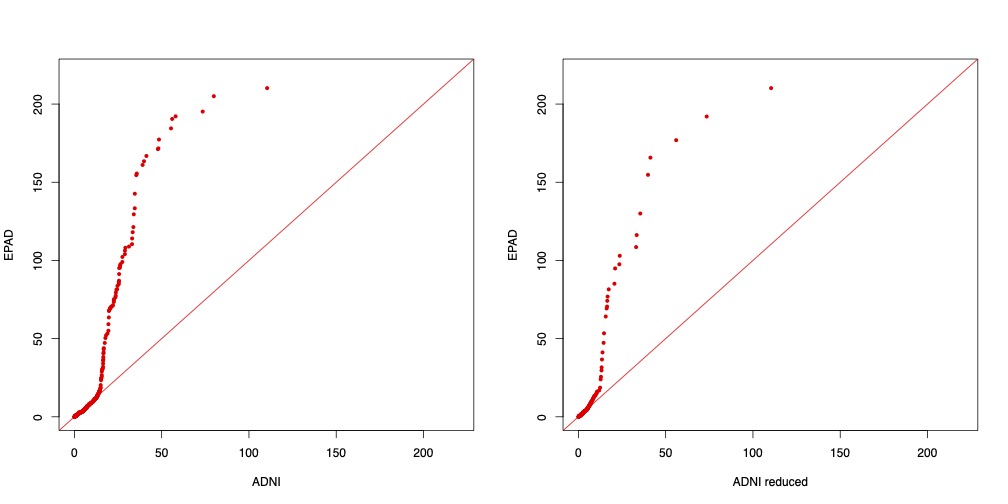


Figure C: Q-Q plots of the distributions of WHML of either ADNI or ADNI reduced dataset vs EPAD.
