# Supplementary material for: Identification of preclinical dementia according to ATN classification for stratified trial recruitment: A machine learning approach
Source: PLoS One. 2023 Oct 19;18(10):e0288039. doi: 10.1371/journal.pone.0288039 (PMC10586674; doi:10.1371/journal.pone.0288039)
Supplement: Attachment — Submitted filename: PONE-D-22-31142_review_01.docx [file pone.0288039.s002.docx]

Thanks for the opportunity to review your paper.

This is an interesting paper that looks that builds on their previous work by replacing logistic regression with machine learning methods and applying feature selection methods to identify preclinical AD state based on the ATN framework/biomarkers. Results were externally validated using ADNI data.

Comments:

- Abstract
  - Methods: “Using machine learning (ML) we identified 13 significant features separating each 30 neurodegeneration-related group from controls (A-T-(N)-)” – This is partially a result as well as a method.
- Introduction
  - Line 45: “was proposed”?
  - This Introduction seems to be very focused on the authors’ previous work with regression modelling as well as the potential of such methods to help targeted recruitment via, as described in the manuscript, “internet-based brain health volunteer research registers such as the Brain Health Registry [5] or the Dementias Platform UK Great Minds register.” These are specific applications related to future directions later described. Including broader context of other work that has been done would improve the Introduction.
- Methods
  - Study design and aims: some of this section feels more like results than aims/design such as “by applying feature selection methods we 75 identified additional significant factors”. I this could be improved by reframing as actual aims and design.
  - Lines 84-86: Here the sample size is 927, but in your previous work (citation #7) is N=1010. Can you explain the discrepancy and what you mean by the sample sizes “matching”.
  - Line 163: You describe WOE. It would be helpful to also describe IV.
  - Line 166-167: Can you provide more clarification for the reader for how IV and WOE are calculated other than simply referencing 26,27,28?
  - Constitutional factors: Did you consider race? I imagine this was a mostly White sample. Can you please report the racial makeup of the sample used? This could be considered another limitation of the study.
  - Line 183: Does the second method (following RandomForest) have a name or can be describe in addition to the citation?
  - Line 189: You explain that you derive 4 models comparing ATN groups vs controls. Is there a reason that you did this rather than use a multiclass classifier?
  - Line 201: Please clarify what you mean by “best”
- Results
  - Most of the comparisons/models are performed on unbalanced samples and AUCs are often sensitive to class imbalance (especially in the case of normal vs AD & concomitant non-AD changes—in that case 525 vs 29). Was this considered during cross-validation (using, for example, stratified cross-validation)?
- Discussion/Limitations/Future Directions/Conclusion
  - The Discussion does a fine job of summarizing and interpreting results from the work of the authors, but it does not contextualize the results within the large amount of work that has been and is being done both other researchers in the field. I think this needs a significant update.
  - In Validation Procedures subsection as well as the Limitations, I think it needs to be more clearly pronounced that the validation is not EPAD-derived models used on ADNI, but features selected on EPAD were used in the training of models on ADNI.
- Figures and Tables
  - Table 1: Could you statistically compare and report p-values for differences between, say, EPAD and ADNI as well as EPAD and ADNI-reduced? It appears there are differences in the sex, family history, APOE4, etc. makeup of the samples, but I am wondering if they are statistically significant.
  - Table 3: It would be helpful if you could clarify how you quantitatively compared models and determined which models were similar or outperformed other models.
  - Figures 1 and 2 are nice.
- Citations
  - Citations 16-18 are not informative enough for someone to know where to find the CSF cutoffs they reference.

Thank you for the opportunity to read your very interesting manuscript and for the contribution your work will give to the field of Alzheimer’s disease research. It was a pleasure to read your work.
