# Supplementary material for: Identification of preclinical dementia according to ATN classification for stratified trial recruitment: A machine learning approach
Source: PLoS One. 2023 Oct 19;18(10):e0288039. doi: 10.1371/journal.pone.0288039 (PMC10586674; doi:10.1371/journal.pone.0288039)
Supplement: Attachment — Submitted filename: Response to Reviewers v2.docx [file pone.0288039.s003.docx]

Dear Dr Ginsberg,

Many thanks for offering us the opportunity to submit a revised version of our manuscript. We note that Reviewer 1 made no suggestions for further improvement in the paper. Reviewer 2 in contrast raised a number of issues which we have addressed point by point below. Please note that these have not translated into major updates to the paper itself as Reviewer 2 comments were mostly questions to the authors which we have hopefully addressed in a satisfactory manner.

We are grateful to both Reviewers for their thoughtful comments and remain available for further discussion as required.

Reviewer1:

1. Is the manuscript technically sound, and do the data support the conclusions?

The manuscript appropriate using techniques and draws conclusions from results.

Author comments: Thank you.

2. Has the statistical analysis been performed appropriately and rigorously?

Statistical and ML analysis was appropriately used. Additional metrics could have been calculated and reported to provide additional insight but the authors did explain they were focusing on AUC. Also, additional techniques could have been used, such as methods specific to unbalanced datasets.

Author comments: Many thanks. We agree further metrics could have been used to provide further details but we believe that the current level of granularity is appropriate for a paper targeting non-ML specialists.

3. Have the authors made all data underlying the findings in their manuscript fully available?

Data used was from EPAD and ADNI that can be requested by researchers.

Author comments: This is correct – EPAD and ADNI are freely accessible to researchers.

4. Is the manuscript presented in an intelligible fashion and written in standard English?

I have no concerns regarding writing.

Author comments: Thank you.

Reviewer 2:

1. In this study, machine learning algorithms for ATN classification only slightly outperformed the logistic regression model (2.2% to 8.3%). What is the effect size?

Author comments: : In our work we applied ML methods for 4 binary classifications and therefore we used the industry-standard AUC metric to demonstrate the effectiveness of our methods, moreover AUC was also used in the original paper (Calvin et.al. [7]). Effect size is typically used to measure the effectiveness of the tests in classical statistical analyses, some examples being Cohen’s d (in the case of t-test), Cohen’s h (in the case of test of proportions) and odds ratio (in the case of logistic regression). However, these are not applicable for binary classifications and in such cases the diagnostic odds ratio (DOR) is typically used instead. Another advantage of AUC is that it shows the overall quality of the model, and DOR can only be calculated given a threshold for separating the two classes. We have not included DOR in our work as it is not well defined for our comparisons, because it is equal to infinity, as for the optimal separation thresholds there are zero False Positives or False Negatives (that are in the denominator of DOR). This is either due to the methods performing really well (as in the case of the most important comparison, Alzheimer’s pathologic change vs Normal AD biomarkers) or the test sets being too small, resulting again in zeroes in the off-main-diagonal elements (FN, FP) of the confusion matrices and thus zero denominators. Therefore, standard metric used to evaluate the model quality in the case of binomial classification is not well defined and because of that we have opted to use the industry-standard AUC.

While we appreciate that numerically the increase in performance may seem marginal, we would like to point out that there is very little practical room for improvement on the previous results. For example, for the most important comparison, Alzheimer's pathologic change vs Normal AD biomarkers, we mention (lines 264-265) that the original results have “already well-performing AUC scores” at about 89%. Improving to about 91% (as we mention in lines 321-322) “bridges almost 20% of the gap to the theoretically possible maximum” of 100%. Furthermore, AUC scores in the mid-90% are not feasible for real-world datasets of this size, as we are reporting on a complex condition (Alzheimer’s disease). In contrast, AUC scores of mid-90% appear only in much larger datasets, or in well-curated or generated datasets used for teaching introductory ML. As such, the theoretical way to further marginally improve the AUC scores towards the mid-90% is to have larger dataset (line 372), or to have other features available in the dataset (lines 343–347, 379-381).

2. In 275-276 lines, “Adding additional 6 features to the random forest models led to extra improvements between approximately 0.8% and 3.5% in AUC”, whether the addition of 6 characteristics, which only improved the model by 0.8% and 3.5%, will lead to model overfitting.

Author comments: We would like to respectfully point out that overfitting is not a consideration in a well-designed ML analysis. In the original version of the paper we had a relevant statement about the applicability of overfitting concerns (lines 185-188), but did not include a reference. We have now rectified this, referring toto the seminal ESL book [34] immediately after that statement and we have added the relevant page numbers for extra clarification.

In principle, overfitting may be an issue when the same data is used for both model creation and evaluation and in such cases it would become apparent when the model is fitted to another dataset. The ML process includes a safeguard against overfitting by using k-fold cross-validation. Specifically, each of the k models are derived on a training set and evaluated on a test set that is non-overlapping with the respective training set. The results (e.g., AUC scores) are reported on the test sets, rather than the training sets. This process ensures the lack of overfitted results by design.

3. Why not apply nonlinear support vector machine multilayer perceptron to compare their prediction ability,

Author comments: : We tried SVM with various kernels (lines 184-185), including non-linear ones. In the article show that the linear kernel outperformed the non-linear ones. There was a good linear separation between the classes and therefore we decided against all possible non-linear SVM methods. But most importantly, the non-linear SVM multilayer perceptron would derive unstable models through the large ratio of number of parameters to dataset size. That is also the reason why we did not try methods based on neural networks (also perceptron-based methods).

4. What the operation used in each iteration to selected the combinations of features and hyperparameters for each group and type of model

Author comments: We deployed a rather standard ML approach. The most informative features (that were subsequently included in the models) were selected using Information Value and Weight of Evidence (line 164). The hyperparameters were selected using a two-step process (lines 200-203), grid search on the parameter space with 1000 iterations, and then increasing the iterations to 10000 when using only the top 100 combinations of hyperparameters.
