# Supplementary material for: Identification of preclinical dementia according to ATN classification for stratified trial recruitment: A machine learning approach
Source: PLoS One. 2023 Oct 19;18(10):e0288039. doi: 10.1371/journal.pone.0288039 (PMC10586674; doi:10.1371/journal.pone.0288039)
Supplement: Attachment — Submitted filename: Response to reviewers 3.docx [file pone.0288039.s004.docx]

Dear Professor Ginsberg,

Thank you for the opportunity to provide revisions to our paper in accordance with a further peer review.

Please see our point-by-point response to the issues raised by the reviewer.

Sincerely Yours

Dr Ivan Koychev

Senior Clinical Researcher

University of Oxford

Reviewer 1: Thanks for the opportunity to review your paper.

This is an interesting paper that looks that builds on their previous work by replacing logistic regression with machine learning methods and applying feature selection methods to identify preclinical AD state based on the ATN framework/biomarkers. Results were externally validated using ADNI data.

Comments:

ABSTRACT
o Methods: “Using machine learning (ML) we identified 13 significant features separating each 30 neurodegeneration-related group from controls (A-T-(N)-)” – This is partially a result as well as a method.

*Authors’ response: Indeed, this sentence contained some of our results. We rephrased it to contain only the class of methods and their purpose.*

INTRODUCTION
o Line 45: “was proposed”?

*Authors’ response: This has been corrected now.*

o This Introduction seems to be very focused on the authors’ previous work with regression modelling as well as the potential of such methods to help targeted recruitment via, as described in the manuscript, “internet-based brain health volunteer research registers such as the Brain Health Registry [5] or the Dementias Platform UK Great Minds register.” These are specific applications related to future directions later described. Including broader context of other work that has been done would improve the Introduction.

*Answer: The reason why we focused on our existing work and the related applications is because the aim of the study was to improve specifically our previous analysis which was based on regression. We have amended the introduction to give a succinct overview of the amyloid prediction literature in particular.*

METHODS
o Study design and aims: some of this section feels more like results than aims/design such as “by applying feature selection methods we 75 identified additional significant factors”. I this could be improved by reframing as actual aims and design.

*Authors’ response: We have amended the Study Design and Aims section to reflect that it referred to our planned analysis.*

o Lines 84-86: Here the sample size is 927, but in your previous work (citation #7) is N=1010. Can you explain the discrepancy and what you mean by the sample sizes “matching”.

*Answer: Indeed, the original paper mentions 1010 as sample size and does not reference that it is reduced to 927 explicitly. However the corresponding Table 4 shows the actual sample sizes of the groups that were used for the analysis; the reason for the lower sample size was the extent of missing data. The group sample sizes are identical to this paper and add up to 927. “Matching” was not referring to a statistical procedure, we have replaced it with “identical”.*

o Line 163: You describe WOE. It would be helpful to also describe IV.

*Authors’ response: Please see the answer of the following suggestion.*

o Line 166-167: Can you provide more clarification for the reader for how IV and WOE are calculated other than simply referencing 26,27,28?

*Authors’ response: We have added more explanations about the design and heuristics of IV and WoE, separated the citations for the two procedures and have added the section of the references that discusses them. However, we feel that going into much deeper discussion would be out of scope of this paper, moreover these procedures are well known and well described in the references.*

o Constitutional factors: Did you consider race? I imagine this was a mostly White sample. Can you please report the racial makeup of the sample used? This could be considered another limitation of the study.

*Authors’ response: The current analysis aimed to determine the extent to which ML can improve on the regression based analyses that were originally carried out in the EPAD study. EPAD is unfortunately homogenous in terms of race and so it was not a factor that we could assess in our original analysis. This translated into the current paper. We appreciate however that ethnicity is a potential factor that may influence ATN status and have added this as a limitation while providing the racial distribution of the two cohorts. The exact numbers of participants, as reported in the databases, are given below (we have added summaries for both datasets in the manuscript):*

| EPAD: |  |  |  |  |  |  |
| --- | --- | --- | --- | --- | --- | --- |
| No info | Asian | Black | Caucasian / white | Latin American | Mauricienne | Moroccan |
| 136 | 2 | 1 | 785 | 1 | 1 | 1 |
|  |  |  |  |  |  |  |
| ADNI: |  |  |  |  |  |  |
| Am Indian / Alaskan | Asian | Black | Hawaiian / Other PI | More than one | Unknown | White |
| 2 | 13 | 23 | 2 | 11 | 2 | 656 |

o Line 183: Does the second method (following RandomForest) have a name or can be describe in addition to the citation?

*Authors’ response: That citation does not refer to a further method, but also to random forest; we have changed the notation to make this more clear. We have also added the names of the other algorithms, rather than using just the names of their implementations.*

o Line 189: You explain that you derive 4 models comparing ATN groups vs controls. Is there a reason that you did this rather than use a multiclass classifier?

*Authors’ response: The original purpose of the paper was to show that ML models have an advantage over more traditional ones, as the logistic regression described in Calvin et al. by using comparable dataset and the AUC metric used in the original paper. Although there are similar metrics for multiclass classifiers, they are not comparable with AUC. We are certainly interested in trying multiclass methods and we consider applying in our follow-up work, although we feel that the class imbalance (discussed in another question) and class sizes would play a role too as the smallest classes would be an even smaller portion of the total count.*

o Line 201: Please clarify what you mean by “best”

*Authors’ response: these are the 100 combinations that result in the largest AUC values. We have clarified this in the text.*

RESULTS
o Most of the comparisons/models are performed on unbalanced samples and AUCs are often sensitive to class imbalance (especially in the case of normal vs AD & concomitant non-AD changes—in that case 525 vs 29). Was this considered during cross-validation (using, for example, stratified cross-validation)?

*Authors’ response: Thank you for highlighting the issue of unbalanced classes. Indeed, we used stratified 5-fold cross validation and we have amended the text to indicate it. This allowed us to have stable AUC values (small standard deviation, as shown on Figure 1) for the 5 folds of the most important EPAD comparison - AD vs Normal AD biomarkers and two of the other EPAD comparisons that also have class imbalances, and also (Figure 2, bottom right pane) for the Non-AD pathologic change vs Normal AD biomarkers comparison for ADNI that has class imbalance (two of the other ADNI comparisons practically don’t have class imbalances, and one of the classes, with n=2, is too small for any reasonable analysis). On the other hand, even using stratified cross-validation resulted in unstable results for the AD and concomitant non-AD vs Normal AD biomarkers comparison on the EPAD data. This is because in addition to the severe unbalance, also the size of one of the classes is too small; that resulted in the discretization shown on Figure 1 and therefore in the instability of the results.*

• Discussion/Limitations/Future Directions/Conclusion
o The Discussion does a fine job of summarizing and interpreting results from the work of the authors, but it does not contextualize the results within the large amount of work that has been and is being done both other researchers in the field. I think this needs a significant update.

*Authors’ response: We agree that the original version of this section could benefit from a broader exploration of the predictive models in the field. The bulk of the work concerns amyloid positivity which is closely aligned with our Alzheimer’s disease and Alzheimer’s pathologic change models. We have amended the relevant section accordingly.*

o In Validation Procedures subsection as well as the Limitations, I think it needs to be more clearly pronounced that the validation is not EPAD-derived models used on ADNI, but features selected on EPAD were used in the training of models on ADNI.

*Authors’ response: Indeed, this is a departure from the more commonly used model validation. We have added extra sentences to the Limitations section to emphasise this.*

FIGURES AND TABLES
o Table 1: Could you statistically compare and report p-values for differences between, say, EPAD and ADNI as well as EPAD and ADNI-reduced? It appears there are differences in the sex, family history, APOE4, etc. makeup of the samples, but I am wondering if they are statistically significant.

*Authors’ response: Below are the tables with the requested p-values. Most of the p-values are significant. The few non-significant p-values are highlighted.*

*Table 1 P-values for comparison of EPAD vs ADNI and EPAD vs ADNI reduced – in yellow are the non-statistically significant comparisons*

| Variable | EPAD vs ADNI | EPAD vs ADNI reduced | Test type |
| --- | --- | --- | --- |
| Numerical |  |  |  |
| Education (years) | 1.41e-24 | 3e-21 | t-test without assumption for equal variances |
| Age (years) | 1.51e-91 | 8.1e-52 | t-test without assumption for equal variances |
| BMI | 1.45e-07 | 0.000186 | t-test without assumption for equal variances |
| MMSE total | 2.22e-28 | 0.0219 | t-test without assumption for equal variances |
| RBANS semantic fluency | 6.4e-15 | 0.0507 | t-test without assumption for equal variances |
| White matter lesion volume | 1.31e-08 | 2.57e-08 | t-test without assumption for equal variances |
| Systolic blood pressure | 0.0589 | 0.675 | t-test without assumption for equal variances |
| Binary |  |  |  |
| Female | 5.96e-06 | 0.102 | chi-square test |
| APOE4 (carrier) | 0.00691 | 0.00616 | chi-square test |
| Dementia family history (yes) | 1.15e-09 | 5.81e-07 | chi-square test |
| Categorical |  |  |  |
| Smoking score (ever smoked) Framingham I | 7.38e-25 | 1.17e-10 | chi-square test |

*Table 2 P-values for comparison of EPAD vs ADNI and EPAD vs ADNI reduced – in yellow are the non-statistically significant comparisons. The second and the third columns are without the assumption of equal variance, while the fourth and the fifth columns present the p-values from t-tests with assumption of equal variance.*

| Variable | EPAD vs ADNI | EPAD vs ADNI reduced | EPAD vs ADNI | EPAD vs ADNI reduced | Test type |
| --- | --- | --- | --- | --- | --- |
|  |  | | Assumption: Equal variance  Equivalent to ANOVA | |  |
| Education (years) | 1.41e-24 | 3e-21 | 9.18e-23 | 5.7e-15 | t-test |
| Age (years) | 1.51e-91 | 8.1e-52 | 2.75e-94 | 5.01e-56 | t-test |
| BMI | 1.45e-07 | 0.000186 | 5.86e-08 | 2.19e-05 | t-test |
| MMSE total | 2.22e-28 | 0.0219 | 3.57e-33 | 0.0297 | t-test |
| RBANS semantic fluency | 6.4e-15 | 0.0507 | 2.43e-15 | 0.0492 | t-test |
| White matter lesion volume | 1.31e-08 | 2.57e-08 | 3.3e-07 | 0.000805 | t-test |
| Systolic blood pressure | 0.0589 | 0.675 | 0.0599 | 0.683 | t-test |

o Table 3: It would be helpful if you could clarify how you quantitatively compared models and determined which models were similar or outperformed other models.

*Answer: All comparisons were done in terms of AUC, all comparisons were done up to the second digit after the decimal point when rounding up to the fourth. We have added that clarification in the legend of that table*.

o Figures 1 and 2 are nice.

*Authors’ response: Thank you.*

CITATIONS
o Citations 16-18 are not informative enough for someone to know where to find the CSF cutoffs they reference.

*Authors’ response: It appears that the URLs of the documents had not been added to the citations, although they appear in our reference database. We have made sure that the URLs appear in the citations and we have added the common section name, clinical values, to each of them.*

Thank you for the opportunity to read your very interesting manuscript and for the contribution your work will give to the field of Alzheimer’s disease research. It was a pleasure to read your work.

*Authors’ response: Thank you for the kind words and the insightful comments and suggestions, they have indeed enhanced the quality of our work.*
